# A Triple Co-Culture Model of the Human Respiratory Tract to Study Immune-Modulatory Effects of Liposomes and Virosomes

**DOI:** 10.1371/journal.pone.0163539

**Published:** 2016-09-29

**Authors:** Rebecca A. M. Blom, Silvia T. Erni, Kristína Krempaská, Olivier Schaerer, R. Maarten van Dijk, Mario Amacker, Christian Moser, Sean R. R. Hall, Christophe von Garnier, Fabian Blank

**Affiliations:** 1 Respiratory Medicine, Department of Clinical Research, Bern University Hospital, University of Bern, Bern, Switzerland; 2 Graduate School for Cellular and Biomedical Sciences, University of Bern, Bern, Switzerland; 3 Mymetics SA, Epalinges, Switzerland; 4 Swiss Federal Institute of Intellectual Property, Bern, Switzerland; 5 Institute of Anatomy, University of Zürich, Zürich, Switzerland; 6 Institute of Human Movement Sciences and Sport, Department of Health Sciences and Technology, ETH Zürich, Zürich, Switzerland; 7 Division of Thoracic Surgery, Bern University Hospital, University of Bern, Bern, Switzerland; 8 Department of Clinical Research, University of Bern, Bern, Switzerland; University of Alabama at Birmingham, UNITED STATES

## Abstract

The respiratory tract with its ease of access, vast surface area and dense network of antigen-presenting cells (APCs) represents an ideal target for immune-modulation. Bio-mimetic nanocarriers such as virosomes may provide immunomodulatory properties to treat diseases such as allergic asthma. In our study we employed a triple co-culture model of epithelial cells, macrophages and dendritic cells to simulate the human airway barrier. The epithelial cell line 16HBE was grown on inserts and supplemented with human blood monocyte-derived macrophages (MDMs) and dendritic cells (MDDCs) for exposure to influenza virosomes and liposomes. Additionally, primary human nasal epithelial cells (PHNEC) and EpCAM^+^ epithelial progenitor cell mono-cultures were utilized to simulate epithelium from large and smaller airways, respectively. To assess particle uptake and phenotype change, cell cultures were analyzed by flow cytometry and pro-inflammatory cytokine concentrations were measured by ELISA. All cell types internalized virosomes more efficiently than liposomes in both mono- and co-cultures. APCs like MDMs and MDDCs showed the highest uptake capacity. Virosome and liposome treatment caused a moderate degree of activation in MDDCs from mono-cultures and induced an increased cytokine production in co-cultures. In epithelial cells, virosome uptake was increased compared to liposomes in both mono- and co-cultures with EpCAM^+^ epithelial progenitor cells showing highest uptake capacity. In conclusion, all cell types successfully internalized both nanocarriers with virosomes being taken up by a higher proportion of cells and at a higher rate inducing limited activation of MDDCs. Thus virosomes may represent ideal carrier antigen systems to modulate mucosal immune responses in the respiratory tract without causing excessive inflammatory changes.

## Introduction

With its ease of access, the vast surface area and extended network of dendritic cells (DCs), the respiratory tract represents a promising target for inhaled immune-modulatory approaches by bio-mimetic nanocarriers such as virosomes and liposomes [1]. Ongoing exposure to environmental air and inhaled antigens represents a continuous challenge to the immune system [1]. Epithelial cells with tight junctions, DCs, macrophages, surfactant film and the muco-ciliary escalator together constitute a structural and functional barrier to protect the respiratory system against harmful pathogens [2].

DCs are the most effective antigen-presenting cells (APCs) found in the respiratory tract that capture, process and present antigens [3,4]. DCs maintain lung homeostasis by orchestrating host responses to benign and harmful foreign substances, making them suitable targets for immunotherapeutic approaches [4]. DCs have the ability to migrate to draining lymph nodes where they stimulate antigen specific proliferation of naïve T-lymphocytes and their differentiation into effector T cells [5]. This provides an important role in allergic airway diseases such as allergic asthma, which is frequently characterized by a skewed Th2 cell response.

Besides DCs, other APCs are present in the respiratory tract including B cells and macrophages [6,7]. Macrophages are scavenger cells that phagocytose particulate antigens and pathogens [8]. Recruitment of macrophages to the site of inflammation can induce both, positive feedback by producing pro-inflammatory cytokines such as IL-1β and tumor necrosis factor alpha (TNF-α) recruiting T cells by chemokine production, or negative feedback by secreting IL-6 and nitric oxide (NO) which dampen DC development and maturation [9]. Depending on the way macrophages are activated, these cells can behave in different ways [10]. The so-called conventional macrophage type 1 phenotype (M1) is activated by IFN-γ or danger signals such as LPS and is characterized as pro-inflammatory. Moreover, it is involved in pathogen killing and plays a role in chronic inflammatory states. Conventional macrophage type 2 phenotype (M2) is alternatively activated by IL-4 and/or IL-13 and is known to have anti-inflammatory properties. In addition, it plays a role in tissue repair, promotes immunoregulation and constructive tissue remodelling[11][12][13]. However, there is no unique definition of M1 or M2 macrophages as clarified in Murray et al [12], therefore we will state how we polarized macrophages and hence call them M1 (IFN-γ and LPS induced) or M2 (IL-4 induced).

Airway epithelial cells (AECs) provide not only barrier function but also play an important role in maintenance of pulmonary homeostasis [14]. Given the close proximity of AECs to airway mucosal dendritic cells (AMDCs) and alveolar macrophages, it is not surprising that AECs regulate innate and adaptive immunity in the lung. AECs modulate the function of immune responsive cells, such as AMDCs, by expressing different surface molecules and secreting cytokines [15,16]. Direct and indirect interactions between AECs and AMDCs seem to play an important role in allergic airway diseases such as allergic asthma, as both contribute to sensitization, initiation and progression of chronic disease [15]. Activated AECs secrete Th2-trophic cytokines such as IL-25 and IL-33 which condition DCs to produce IL-5 and IL-13, and thereby favour Th2 polarization [15,17].

Bio-mimetic antigen nanocarriers such as virosomes or liposomes are promising compounds for inhaled, non-invasive, and specifically tailored immune-modulation. Influenza virosomes and liposomes are spherical vesicles, comprised of lipids and close to 100 nm in diameter. Not only do virosomes provide carrier function by incorporating or encapsulating antigens, but they additionally are potent immune stimulators due to influenza virus envelope proteins integrated in the membrane [18,19]. Also, influenza virosomes provide high safety and excellent tolerability due to absence of genetic material and their purity, having already been approved for human use in prophylactic applications [20–22] and tested clinically in various diagnostic and therapeutic approaches[23].

The aim of this study was to establish a human model of the respiratory tract to study the interplay between epithelial cells, macrophages, and DCs, as well as interactions with bio-mimetic nanocarriers such as liposomes and virosomes to closely represent the human situation *in vitro* and therefore reduce animal experimentation to a minimum. To achieve this, we optimized a triple co-culture model integrating a human bronchial epithelial cell line (16HBE), monocyte-derived macrophages (MDMs) and monocyte-derived DCs (MDDCs)[24]. In addition, uptake and immune effects of influenza virosomes or liposomes were tested with *in vitro* differentiated primary human nasal epithelial cells (PHNEC) and EpCAM^+^ epithelial progenitor cell mono-cultures representing cells of the larger and smaller airways, respectively which are key players of the pulmonary innate immune system to inhaled pathogens.

## Material and Methods

### Atto647-PE Conjugation

The fluorchrome Atto647N NHS ester (1.7 μmol in DMSO; Sigma) was conjugated to 8.5 μmol OPPE (1-Oleoyl-3-palmitoyl-rac-glycero-2-phosphoethanolamine; Bachem, Bubendorf, Switzerland) in 90 mM dodecyl octaethylene glycol ether (OEG; Sigma)-PBS pH 7.4.

### Virosome and Liposome Formulation

Influenza virosomes were prepared as follows. In short: per ml of final formulation, 8 mg of DOPC (1,2-dioleoyl-sn-glycero-3-phosphocholine; Merck, Darmstadt, Germany) and 1 mg of OPPE were dissolved in 100 mM OEG in PBS pH 7.4 (52.7 mM phosphate, 82 mM NaCl). Inactivated influenza A/Brisbane/59/2007 H1N1 virus was mixed with PBS and centrifuged at 100.000xg for 1h at 18°C. The pellet of inactivated influenza virus was resuspended with 100 mM OEG-PBS pH 7.4 for 10 min followed by sonication for 1 min at 30°C. This mixture was centrifuged at 100.000xg for 1h at 18°C to pellet down the nucleocapsid complex. The supernatant containing the solubilized influenza membrane proteins and lipids was used for virosome formulation and mixed with phospholipids at a concentration of 0.2 mg/ml hemagglutinin (HA). Virosome formation took place after removal of OEG detergent using 0.375 g per ml of formulation of SM2 Bio-Beads (BioRad) twice for 1h and once for 30 min at room temperature whilst mixing at 100 rpm. Fluorescent virosomes were obtained by adding Atto647-PE as indicated prior to detergent removal to enable peptide incorporation. Liposomes were prepared similarly by leaving out the influenza virus component but following the same procedure. At the end of the process virosomes and liposomes were sterile filtered on 0.22 μm units (Millex-GP; Merck Millipore).

### Characterization of Virosome and Liposome Formulations

Nanocarriers were thoroughly characterized prior to use. Particle size, homogeneity, as well as the amount of HA were analyzed. Size determination was performed in PBS pH 7.4 by dynamic light scattering (DLS) using a Zetasizer Nano S instrument and by nanoparticle tracking analysis (NTA) on a NanoSight NS300 instrument (both from Malvern Instruments, Malvern, UK). Samples were routinely measured for endotoxin by performing limulus amebocyte lysate (LAL) test.

Influenza HA concentration of virosomes was determined by SDS-PAGE using a 4–20% precast mini-protean TGX gel (BioRad) and Coomassie Brilliant Blue R-250 (BioRad) staining with a One-Color Protein Molecular Weight Marker (Odyssey, LiCor). HA concentration was confirmed by Spotblots and Western Blots using nitrocellulose (0.2 μm pore size; Life Technologies) and rabbit anti-HA and rabbit anti-OVA serum followed by secondary goat-anti-rabbit IRDye 800CW (LiCor Biosciences), visualized and quantified using the LiCor Odyssey imaging system (Lincoln, Nebraska, USA). Selected samples were also quantified in a SRID (single radial immunodiffusion) assay to confirm the HA concentration.

### Human Monocyte-Derived DC Cultures

Cells were obtained from buffy coats of healthy individuals provided by blood donors from the Regional Red Cross Blood Donation Centre (Bern, Switzerland). Peripheral blood mononuclear cells (PBMCs) were obtained by Pancoll density centrifugation and monocytes were isolated by CD14 positive selection using MACS microbeads (Miltenyi Biotec GmbH, Bergisch Gladbach, Germany). MDDCs were generated by culturing in RPMI 1640 medium (Invitrogen) supplemented with 10% fetal calf serum (FCS), 1% L-glutamine (2 mM, Invitrogen), 1% penicillin/streptomycin (100 U/ml, Invitrogen), 10 ng/ml GM-CSF (R&D Systems) and 10 ng/ml IL-4 (R&D Systems) for 7 days at 37°C/5% CO_2_.

### Human Monocyte-Derived Macrophage Cultures

Cells were obtained from buffy coats of healthy individuals provided by blood donors from the Regional Red Cross Blood Donation Centre (Bern, Switzerland). PBMCs were obtained by Pancoll density centrifugation and monocytes were isolated by Monocyte Isolation Kit II (Miltenyi Biotex GmbH). MDMs were generated by culturing in RPMI 1640 medium (Invitrogen) supplemented with 10% FCS, 1% L-glutamine (2 mM, Invitrogen), 1% penicillin/streptomycin (100 U/ml, Invitrogen), 10 ng/ml M-CSF (R&D Systems) for 8 days at 37°C/5% CO_2_. Where indicated, macrophages were incubated with LPS (100 ng/ml) and IFN-γ (20 ng/ml) for 24h to induce M1 or incubated with IL-4 (20 ng/ml) for 24h to induce differentiation into M2 [25].

### Human Epithelial Cell Line

Differentiated SV-40 transformed human bronchial epithelial cell line (16HBE14o- cells) was maintained in MEM 1X, with Earle’s Salts, without L-Glutamine (Gibco BRL Life Technologies Invitrogen AG, Basel, Switzerland) supplemented with 1% glutamine/penicillin/streptomycin (Gibco BRL) and 10% FCS (Gibco BRL). About 5x10^6^ cells were seeded in collagen coated (Pure Col, Purified Bovine Collagen Solution, Advanced Biomatrix, 1:50) T75 flasks (Falcon, US) for further cell culture at 37°C/5% CO_2_.

### Primary Human Nasal Epithelial Cells

Ten healthy adult volunteers were recruited for a brushing of the inferior surface of the middle turbinate of both nostrils in order to obtain airway epithelial cells. This was performed by using a cytological brush (Dent-O-Care, No 720, London, UK) [26]. The study protocol was approved by the Ethics Committee of the Canton of Bern, Switzerland (KEK 77/09). Informed written consent was obtained from all study participants. Cells were grown in Bronchial Epithelial Basal Medium (BEBM, Lonza, Switzerland) supplemented with Single Quots (SQ, Lonza, Switzerland) and seeded in uncoated T25 flasks (Falcon). When reaching 80–100% confluency cells were detached with trypsin (Gibco BRL) and 10^5^ cells were seeded per uncoated insert (0.4 μm pore size, sealed PET capillary pore membrane, 12 well plate; Greiner Bio-One, Germany).

### Air-Liquid Interface (ALI) Cultures

Primary human nasal epithelial cells grown on inserts (as described above) were differentiated in Maintenance medium (PneumaCult-ALI supplemented with 6 μl Maintenance, 3 μl Hydrocortison, 1.2 μl Heparin (Stemcell), 0.9 μl Primorcin (InvivoGen, US)) given to the lower chamber, while the apical side of the epithelial cells remained facing air. Cells were cultured for 8 weeks for full differentiation.

### Human Epithelial Progenitor Cells (EpCAM^+^) and Pericytes (EpCAM^-^)

To prospectively isolate lung pericytes and epithelial cells, we used lung specimens obtained from patients following surgical resection for early stage lung cancer. All patients gave informed written consent for usage of surgical material for research purposes, which was approved by Ethics Commission of the Canton of Bern, CH (KEK-BE:042/2015). Preparation of lung tissue and cell sorting was performed as previously described with modifications [27]. Briefly, normal appearing lung tissue was resected from the tumor foci at a distance > 5 cm and digested using a solution of collagenase I and II (Worthington Biochemical Company). Digestion of lung tissue was halted following the addition of 10% FBS (Invitrogen). Single cells were stained with a panel of fluorescently conjugated human monoclonal antibodies directed at the following epitopes (all eBioscience): CD45-PB, CD14-PB, CD31-PB, CD235a-PB, CD73-APC and CD90-FITC, EpCAM-PE-Cy7. To exclude dead cells, 7-AAD (eBioscience) was added prior to sorting. Proper placement of gates was determined using fluorescence minus one strategy [28,29]. Cells were sorted using a BD FACS Aria III (BD Biosciences, Franklin Lakes, NJ, USA). A fraction of mesenchymal cells simultaneously expressing CD73 and CD90, while lacking the epithelial cell adhesion marker EpCAM and the lineage markers CD45, CD14, CD31 and CD235a were directly cultured in a 6-well tissue culture plate pre-coated with 0.1% gelatin in alpha-MEM (Sigma) supplemented with 1% FBS (Gibco), 20 ng/mL of recombinant human FGF2 (Gibco), 25 ng/mL of recombinant human EGF (Gibco), 1.25 mg of human Insulin (Sigma), 1% pen-strep (Sigma). An epithelial fraction simultaneously expressing EpCAM and CD73 while lacking CD90 and lineage markers were cultured in 6 well plates precoated with a solution of human collagen I (Sigma) and human collagen IV (Sigma) and grown in CNT-PA plus (CELLNTEC, Bern, CH). Cells were grown at 37°C, 5% CO2 and low O_2_ (3%) until reaching confluence and expanded in their respective media.

### Triple Co-Culture

The triple co-cultures were prepared as previously described [30,31]. The 16HBE14o- cell line (initial number of 0.5 x 10^6^ cells) was grown on cell culture inserts (3.0 μm pore size, sealed PET capillary pore membrane, 12 well plate; Greiner Bio-One, Germany) that were previously coated with collagen (Advanced Biomatrix). After three days expansion time, the confluent monolayer of 16HBE cells was supplemented with differentiated MDMs and MDDCs as follows: MDDCs were harvested, washed, centrifuged and resuspended in MEM medium and 2.5x10^5^ cells in 75 μl medium were added to the basal side of the inserts placed upside down on a petri dish. The petri dish with the inserts was covered and placed in the incubator for 1.5h–2h. MDMs were harvested by washing with PBS followed by addition of 380 μl Accutase (Gibco BRL). Plates were placed in the incubator for 5–10 min. Cells were then detached with cell scrapers (Semadeni, Ostermundigen, Switzerland). The activity of Accutase was stopped by adding MEM. A volume of 500 μl containing 1.9x10^5^ cells was added on the apical side of the epithelial monolayer on the insert, forming the upper chamber. After 2h, 750 μl of MEM medium was added to the lower chamber.

### Uptake of Virosomes and Liposomes

To study uptake of virosomes and liposomes, cells of interest were cultured for 18h at 37°C in presence of virosomes, liposomes or controls. One ml of suspended cells was incubated for 18h with virosomes (5 μg HA), liposomes, or control (PBS) in the same dilution as virosomes. Uptake was determined by measuring Atto647 signal by flow cytometry (LSRII, BD Biosciences, Franklin Lakes, NJ, USA). Data was analyzed using FlowJoX (TreeStar, Ashland, OR, USA) software.

### Phenotyping of Cells

MDDC and MDM phenotype was determined by flow cytometry after treatment with virosomes, liposomes or appropriate controls for 18h. One ml of cell suspension was incubated with virosomes (5 μg HA), liposomes in the same dilution, or the following controls: medium only, PBS in the same dilution as virosomes, LPS (100 ng; Sigma) or inactivated influenza virus A/Brisbane (5 μg HA). 16HBE cells were collected by trypsinisation (Gibco BRL), MDMs and triple co-cultures were resuspended by Accutase digestion (Gibco BRL) and by cell scraping. Cells were treated with FcR block on ice followed by viability staining with Fixable Viability Dye eFluor506 (eBioscience, Vienna, Austria) for 30 min on ice. As positive controls, cells heated at 65°C for 15 min or frozen at -80°C for 30 min were used. Unless indicated otherwise, antibodies were purchased from eBioscience and utilised with appropriate isotype controls. For MDMs: CD14-Alexa Fluor 700, CD68-PE-eFluor610, CD163-PerCPeFluor710, CD40-APC-Cy7 (Biolegend, London, UK), HLA-DR-Brilliant Violet 785 (BioLegend), CD86-Brilliant Violet 605 (BioLegend), CD80-Brilliant Violet 421 (BioLegend, CD206-PE-Cy7, CD36-APC-Cy7 (BioLegend). For MDDCs: CD11c-Pe-Cy7, CD1c-Alexa Fluor 700 (BioLegend), CD83-PE-Cy7 (BioLegend), PD-L1-eFluor450, PD-L2-PerCP-eFluor710 and CCR7-APC-eFluor780. For epithelial cells and triple co-cultures additionally: CD209-PE-Cy7 (DC-Sign), CD86-PE and CD326-Brilliant Violet 650 (EpCAM; BioLegend). Flow cytometry was performed by using flow cytometry SORP LSR II (BD Biosciences).

### Intracellular Cytokine Staining

Cells were treated with 20 μg/ml Brefeldin A (eBioscience) to stop protein transport. Subsequently, cells were stained for surface markers as mentioned above. Cells were fixed in 1% formalin solution followed by intracellular staining with the following anti-cytokine antibodies with appropriate isotype controls diluted in permeabilization buffer (PBS + 0.1% saponin + 10% FCS): IL-10-Alexa Fluor 488, IL-12-PE (all eBioscience).

### Cytokine Detection

Medium of the upper chamber of the triple co-cultures and supernatant of 16HBE mono-cultures were collected and production of IL-8, IL-1β and TNF-α was analysed by employing ELISA Kits from R&D Systems (Minneapolis, US) according to the manufacturer’s specifications. Optical density was measured with a microplate reader (Tecan reader) at a wavelength of 450nm.

### Laser Scanning Microscopy

Particle uptake was confirmed using laser scanning microscopy (LSM) to detect Atto647 emission in cells. PHNECs were fixed in 70% ethanol and permeabilized with 0.2% Triton X-100 (Sigma-Aldrich). Cells were stained with primary antibodies mouse anti-β-tubulin (1:100; Life Technologies), rabbit anti-occludin (1:50; Molecular Probes) or rabbit anti-mucin (1:50; Santa Cruz Biotechnology) overnight at 4°C. The following secondary antibodies were used for 1h at room temperature: goat anti-mouse Alexa488 (1:200; Molecular Probes), goat anti-rabbit Alexa546 (1:200; Life Technologies) and phalloidin Atto390 (1:25; ATTO-TEC). Cells were washed and embedded in Aquatex mounting medium (Merck KGaA, Darmstadt, Germany). Optical sections were taken with a Zeiss LSM 710 (Carl Zeiss AG, Feldbach, Switzerland) with a 40x oil objective (Plan-Apochromat 40x/1.40 Oil) and a digital zoom of 1.4x. Image processing was performed using Imaris (Bitplane AG, Zurich, Switzerland) software.

### Statistics

Statistical analyses were conducted using R version 3.2.1 [32]. All graphical representations were prepared using the R package ggplot2 [33]. Differences in measured frequency and mean fluorescence intensity (MFI; median) between groups were tested using an ANOVA. Main effects of treatment (consisting of virosomes, liposome and control where appropriate) and culture type (consisting of mono- or co-culture) were included in the model. Tukey’s honest significant difference (HSD) post hoc test was used to investigate individual paired comparisons. Where shown to be significant, the effect of Atto647 was included as a covariate. Appropriateness of ANOVA models was verified by analysing the residuals. Experiments are all biological repetitions stated in each figure legend.

## Results

### Virosome and Liposome Characterization

Influenza virosomes and liposomes were extensively characterized for particle size, homogeneity, as well as HA content and endotoxin contamination. Particle size was measured by DLS and NTA that routinely provided a hydrodynamic diameter (particle diameter plus water shell) of 90–96 nm, and particle sizes of 84–86 nm for all nanocarrier formulations respectively (Table 1). Nanoparticle quality was consistently uniform and reproducible throughout all experiments performed. Protein concentration of HA was determined by performing SDS-PAGE, Spotblot and Western Blot (data not shown). The HA concentration was consistently around 50 μg/ml (data not shown). In exposure experiments influenza virosomes were further employed at a concentration of 5 μg/ml HA, whereas liposomes and PBS were utilized at the same dilution factor. Routine endotoxin measurements of virosomes and liposomes by LAL test yielded consistent results below the threshold of 10.0 EU/ml in the concentrated formulations (data not shown).

**Table 1:** Characterization of virosomes and liposomes.

Samples	DLS^1^		NTA^2^
	Size ± SD (nm)^3^	PDI^4^	Size ± SD (nm)^5^
Virosome	95.0 ± 0.6	0.03	85.3 ± 0.8
Virosome-Atto647	95.6 ± 0.7	0.04	84.9 ± 0.5
Liposome	90.0 ± 1.7	0.02	85.5 ± 0.8
Liposome-Atto647	90.8 ± 2.5	0.03	84.0 ± 0.4

Influenza virosomes and liposomes with or without conjugated fluorochrome Atto647 were analyzed for particle size by DLS and NTA. One representative measurement from two independent formulations of virosomes and liposomes is shown.

^1^ Dynamic light scattering

^2^ Nanoparticle tracking analysis

^3^ Hydrodynamic diameter

^4^ Poly Dispersity Index

^5^ Modal particle size.

### Treatment with Virosomes or Liposomes Does Not Induce Cell Death

Potential cytotoxic effects were determined prior to analysis of particle uptake and phenotype change. Cells were incubated with virosomes, liposomes or appropriate controls. Cells kept for 15 min at 65°C or stored at -80°C for 30 min served as positive controls and showed a high cell death rate (data not shown). Less than 10% of dead cells were detected in all cell types after exposure to particles compared to PBS treated cells in culture (S1 Fig). The evaluated concentration of virosomes was 5 μg/ml HA, and this was therefore chosen as an optimal concentration for all subsequent experiments. Dead cells were always excluded for further flow cytometry analyses.

### Uptake Efficiency Differs between Virosomes and Liposomes

In order to investigate whether virosomes and liposomes are taken up by different cells of the respiratory tract and to compare their dynamics in mono- and triple co-culture, cells were incubated with virosomes, liposomes or a PBS control. For triple cell co-cultures gating was performed for size, single cells, live cells and split into EpCAM^+^ cells for 16HBE and EpCAM^-^ cells. EpCAM^-^ cells were further divided into DC-Sign^+^ (for MDDCs) and DC-Sign^-^ (for MDMs; S2A Fig). Mono-cultures were gated for single, live cells followed by EpCAM^+^ cells for epithelial cells, CD1c^+^ and CD11c^+^ double positive cells for MDDCs (S2B Fig) and CD14^+^ and CD68^+^ double positive cells for MDMs (S2C Fig).

**Fig 1:**
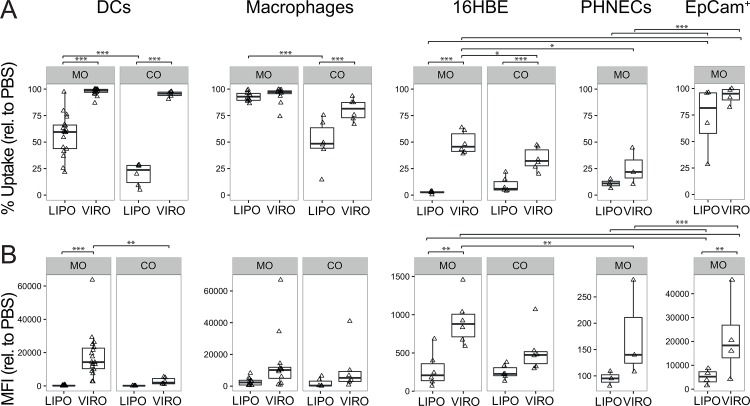
Uptake of virosomes or liposomes in mono- (MO) or co-culture (CO). MDDCs (DCs), MDMs (macrophages), 16HBE, PHNEC and human epithelial progenitor cells (EpCAM^+^) were incubated with either virosomes (VIRO), liposomes (LIPO) or control (PBS) for 18h at 37°C. Uptake of virosomes and liposomes was determined by measuring Atto647 signal by flow cytometry. Frequency **(A)** and MFI **(B)** are shown relative to PBS. Data represents at least four independent experiments. Statistical significance was determined by ANOVA followed by Tukey’s HSD post hoc test to investigate individual paired comparisons. *p<0.05; **p<0.01; ***p<0.001.

In MDDCs uptake of both nanocarrier types was observed with significantly more cells capturing virosomes than liposomes in both mono- and co-culture (p<0.001; Fig 1A). Interestingly, liposomes were taken up by less MDDCs in co-culture compared to mono-culture (p<0.001; Fig 1A). Furthermore, in MDDCs more virosomes than liposomes were taken up per cell in mono-culture as measured by MFI (p<0.001; Fig 1B), whereas significantly less virosomes were taken up per cell in co-culture compared to mono-culture (p = 0.002; Fig 1B).

In co-culture, MDMs showed higher uptake frequency for virosomes than liposomes (p<0.001; Fig 1A). Similar to MDDCs, MDMs in mono-cultures took up significantly more liposomes than in co-cultures (p<0.001; Fig 1A). The amount of particles taken up per cell was similar in mono- and co-culture.

Significantly more 16HBE cells in both mono- and co-culture captured virosomes than liposomes (p<0.001; Fig 1A). Compared to virosome uptake in 16HBE mono-cultures, 16HBE co-cultures showed reduced virosome uptake (p = 0.023; Fig 1A). Regarding particle uptake per cell in 16HBE mono-cultures, a higher MFI was detected for virosomes than liposomes (p<0.01; Fig 1B). Virosome uptake in frequency (p<0.05) and MFI (p<0.01) was higher in 16HBE mono-culture than in PHNEC mono-culture (Fig 1).

Human epithelial progenitor cells (EpCAM^+^) internalized more virosomes than liposomes according to MFI (Fig 1B). Both virosomes and liposomes were taken up by EpCAM^+^ cells with a much higher efficacy than by 16HBE or PHNECs (p<0.001; Fig 1). Matched EpCAM^-^ cells took up both virosomes and liposomes successfully (S3 Fig).

**Fig 2:**
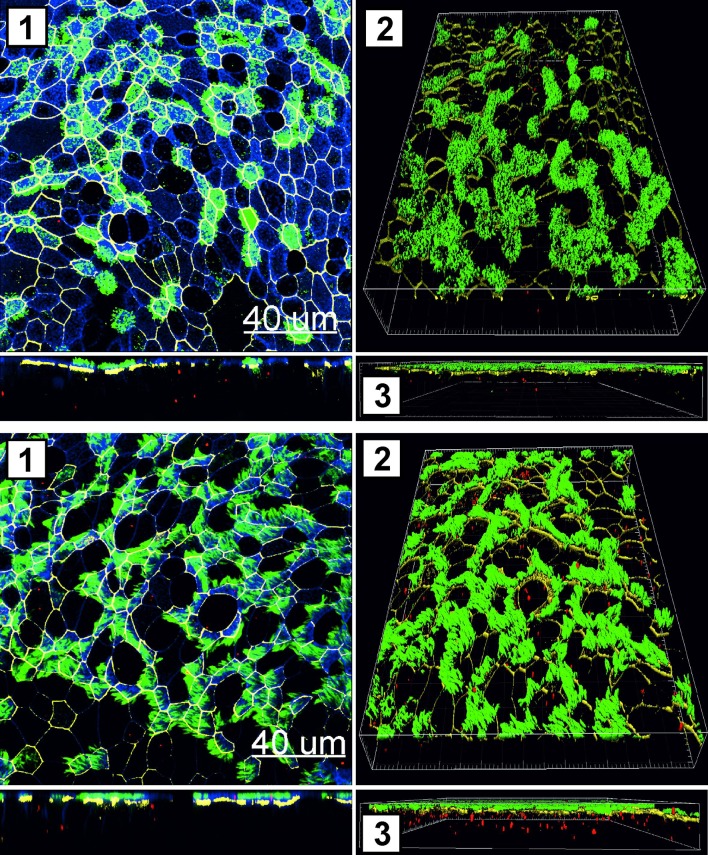
Exposure of virosomes or liposomes to PHNEC ALI culture. Uptake of liposomes **(A)** or virosomes **(B)** following treatment in PHNEC ALI culture was analyzed by LSM. Micrograph was obtained from three-dimensional stacks of consecutive optical sections and analyzed with Imaris software. Green: cilia (β-Tubulin), red: liposomes/virosomes, blue: actin cytoskeleton, white/yellow: tight junctions (occludin). (1) xy-projections (top panel) and xz-projections (lower panel) (2, 3) 3D reconstruction of cilia, virosomes and tight junctions, view from top (2) and view from the side (3). One representative experiment from three independent experiments is shown.

**Fig 3:**
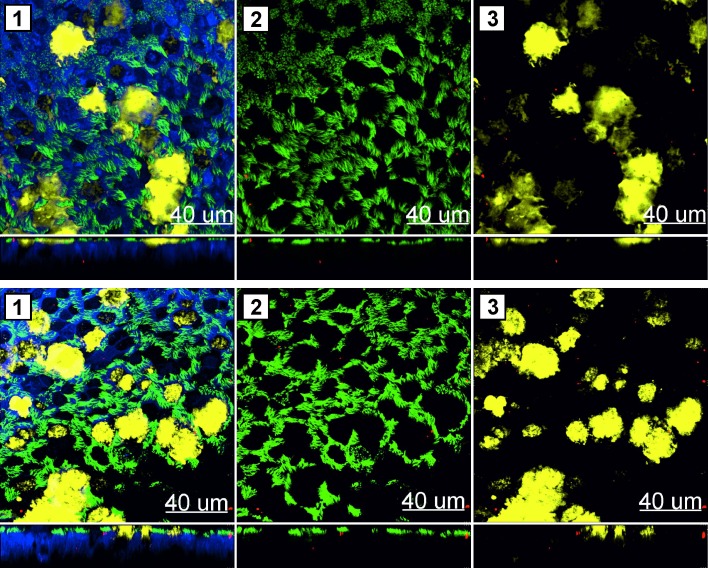
Mucus staining of PHNEC ALI following virosome or liposome exposure. The effect of liposomes (A) or virosomes (B) exposure was analyzed by LSM. Micrographs were obtained from three-dimensional stacks of consecutive optical sections and analyzed with Imaris software. Green: cilia (β-Tubulin), red: liposomes/virosomes, blue: actin cytoskeleton, yellow: mucin. xy-projections (top panel) and xz-projections (lower panel) (1) merged image (2) cilia and liposomes/virosomes (3) mucin and liposomes/virosomes. One representative experiment from three independent experiments is shown.

Particle uptake in PHNEC was confirmed by LSM. Cells were stained for β-Tubulin (cilia), phalloidin (actin cytoskeleton) and either occludin (tight junctions; Fig 2) or MUC5AC (mucin; Fig 3). Virosomes and liposomes were taken up and localised both intracellularly and apically between cilia.

### Characterization of Phenotype and Cytokine Profile after Virosome and Liposome Treatment in Mono- and Co-Cultures

We next investigated whether treatment virosomes or liposomes induce MDMs, MDDCs and 16HBE phenotype changes or activation by expression of co-stimulatory surface molecules in both mono- and co-cultures.

16HBE cells were analysed by flow cytometry for surface expression of HLA-DR, CD40, CD80 and CD86, but no significant changes were detected (S4 Fig).

**Fig 4:**
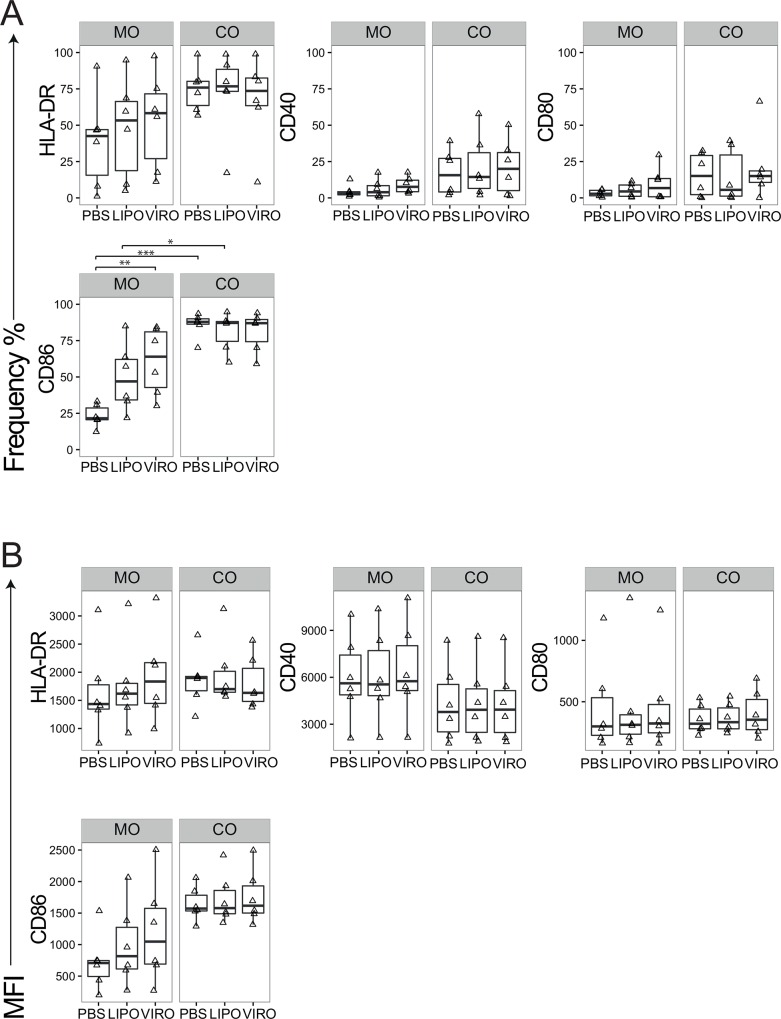
Expression of surface markers in MDDCs upon uptake of virosomes or liposomes. Cells in mono- (MO) or co-culture (CO) were incubated for 18h with either virosomes (VIRO), liposomes (LIPO) or controls (PBS, as shown). Expression of surface molecule markers HLA-DR, CD40, CD80, and CD86 were measured by flow cytometry. Figures show the receptor expression in frequency **(A)** and MFI **(B)** of six independent experiments. Statistical significance was determined by ANOVA followed by Tukey’s HSD post hoc test to investigate individual paired comparisons. *p<0.05; **p<0.01; ***p<0.001.

For MDDCs we analyzed the phenotypic and co-stimulatory markers HLA-DR, CD40, CD80, CD86, CD83, PD-L1, PD-L2, CCR7 and intracellular cytokines IL-10 and IL-12 with respect to their relevant isotype controls. The marker CD86 showed significant upregulation after treatment with virosomes in mono-culture compared to PBS (p<0.01) and in co-culture after PBS (p<0.001) or liposome (p = 0.05) treatment compared to mono-culture (Fig 4). The other markers showed no phenotype change in mono-culture (S5 Fig). To confirm that MDDCs were able to undergo activation, positive controls (LPS, inactivated influenza virus) were employed that showed high expression of surface markers in MDDCs (S7 Fig).

**Fig 5:**
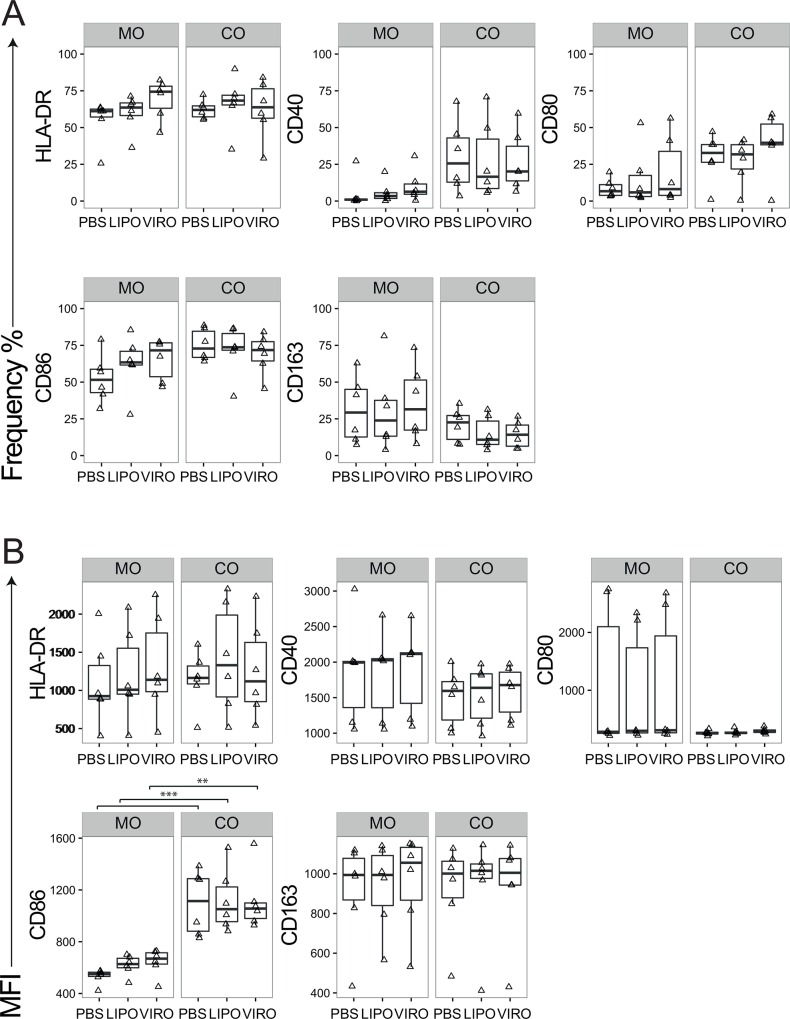
Expression of surface markers in MDMs upon uptake of virosomes or liposomes. Cells in mono- (MO) or co-culture (CO) were incubated for 18h with either virosomes (VIRO), liposomes (LIPO) or controls (PBS, as shown). Expression of surface molecule markers HLA-DR, CD40, CD80, CD86 and CD163 were measured by flow cytometry. Figures show the receptor expression in frequency **(A)** and MFI **(B)** of six independent experiments. Statistical significance was determined by ANOVA followed by Tukey’s HSD post hoc test to investigate individual paired comparisons. *p<0.05; **p<0.01; ***p<0.001.

MDMs were analysed for HLA-DR, CD40, CD80, CD86, CD163, IL-10 and IL-12. The main effect observed was higher CD86 expression per cell in co-culture compared to mono-culture (p<0.01; Fig 5B), whereas no significant differences were detected for the other markers (Fig 5 and S6 Fig). To confirm that MDMs were able to undergo activation, positive controls for MDMs (LPS, inactivated influenza virus) were employed that showed high expression of surface markers (S8 Fig).

**Fig 6:**
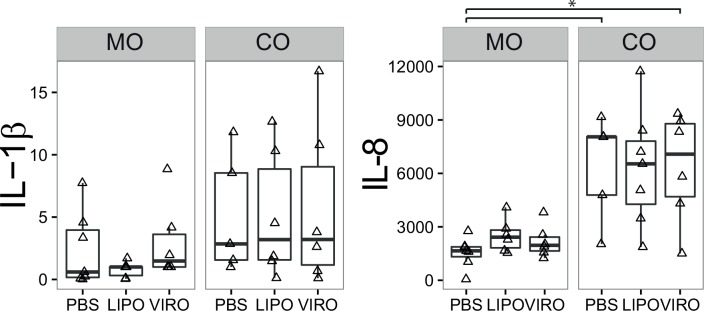
IL-1β and IL-8 secretion in 16HBE cells. Cells in mono- (MO) or co-culture (CO) were incubated for 18h with either liposomes (LIPO), virosomes (VIRO) or controls (PBS, as shown). Supernatants were collected to perform IL-1β and IL-8 ELISA. Data represents six independent experiments. Statistical significance was determined by ANOVA followed by Tukey’s HSD post hoc test to investigate individual paired comparisons. *p<0.05.

Minimal changes in the cytokine profile were observed in 16HBE mono- and co-cultures after treatment with virosomes or liposomes. Although an increase in IL-8 secretion was observed in co-cultures after treatment with virosomes (p = 0.034), the effect was also observed with PBS (p = 0.011) and therefore might not be related to nanocarriers. No significant changes were measured for IL-1β (Fig 6), and levels of TNF-α were below detection limit in all conditions (data not shown).

Taken together, we observed minimal differences between virosome- and liposome-induced phenotypic and co-stimulatory marker expression in different cell types, either in mono- or co-culture. MDDCs overall underwent moderate activation compared to MDMs and 16HBE cells.

### Uptake of Virosomes and Liposomes in M1 and M2 Differentiated Macrophages

**Fig 7:**
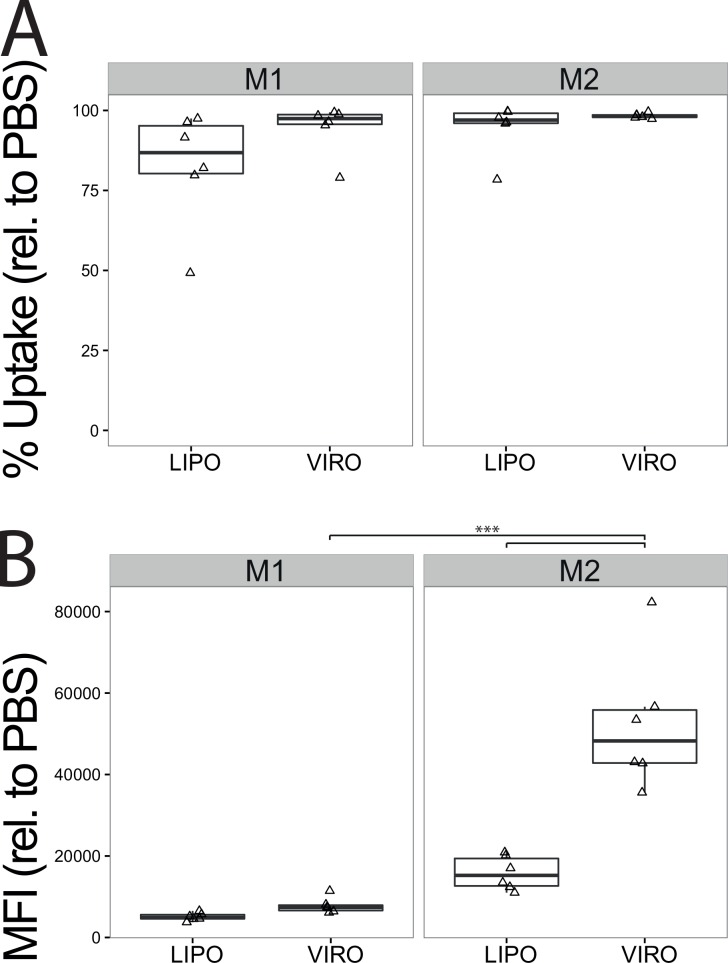
Uptake of virosomes or liposomes by M1 and M2 differentiated macrophages in mono-cultures. Cells in mono-cultures were differentiated for 24h into M1 and M2 type macrophages before being incubated for 18h with either virosomes (VIRO), liposomes (LIPO) or controls (PBS). Uptake of virosomes and liposomes was determined by measuring Atto647 signal by flow cytometry. Frequency **(A)** and MFI **(B)** are shown relative to PBS. Data represents six independent experiments. Statistical significance was determined by ANOVA followed by Tukey’s HSD post hoc test to investigate individual paired comparisons. *p<0.05; **p<0.01; ***p<0.001.

Following polarization into M1 and M2 macrophages we analyzed uptake in these cells for both liposomes and virosomes. There was no significant difference in frequency of virosome or liposome uptake (Fig 7A), but the in MFI was significantly higher for virosomes than liposomes in M2 macrophages (p<0.001; Fig 7B) and significantly more virosomes were taken up by M2 than by M1 cells (p<0.001; Fig 7B). In summary, M2 differentiated macrophages took up more of both bio-mimetic nanocarriers than M1 differentiated macrophages. Also, M2 differentiated macrophages took up significantly more virosomes than liposomes.

### Phenotype of M1 and M2 Differentiated Macrophages after Treatment with Virosomes and Liposomes

**Fig 8:**
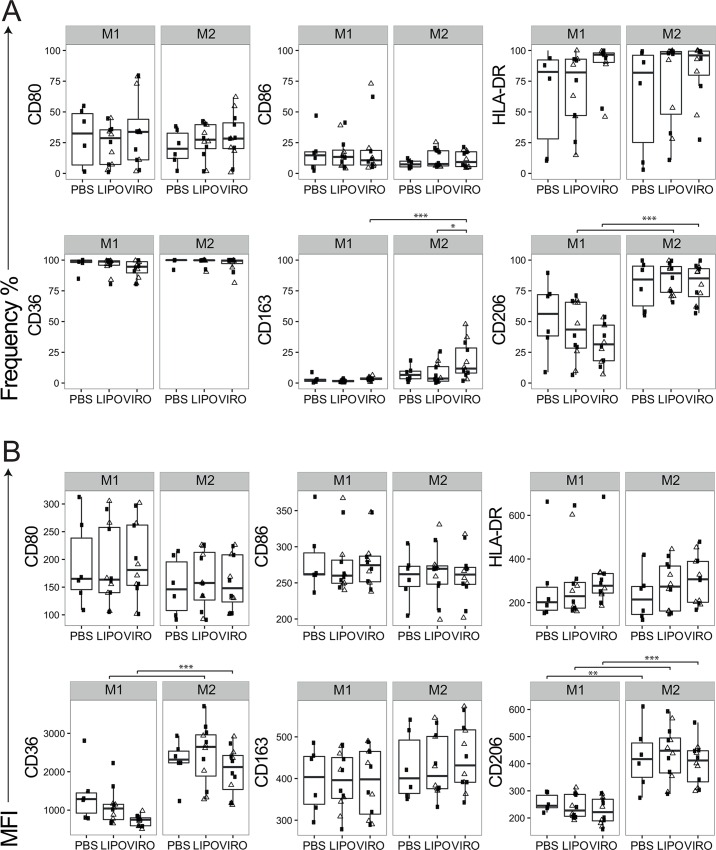
Expression of surface markers in M1 and M2 differentiated MDMs upon uptake of virosomes or liposomes in mono-culture. Cells in mono-culture were differentiated for 24h into M1 or M2 macrophages before being incubated for 18h with either virosomes (VIRO), liposomes (LIPO) or controls (PBS). Expression of surface molecule markers HLA-DR, CD80, CD86, CD36, CD163 and CD206 were measured by flow cytometry. Figures show the receptor expression in frequency **(A)** and MFI **(B)** of six independent experiments. Statistical significance was determined by ANOVA followed by Tukey’s HSD post hoc test to investigate individual paired comparisons. *p<0.05; **p<0.01; ***p<0.001.

MDMs differentiated into M1 and M2 were further analysed for phenotype changes after addition of virosomes or liposomes to detect whether exposure to these nanocarriers is affecting phenotype and function of macrophages that have undergone polarization. *In vitro* differentiation resulted in macrophage populations with a M1 (HLA-DR, CD80, CD86) and M2 phenotype (CD36, CD163, CD206). Following treatment of virosomes and liposomes, the surface phenotypes of both M1 and M2 macrophages remained unchanged (Fig 8). Overall, there was no phenotype change upon virosome or liposome treatment.

## Discussion

In recent years, influenza virosomes have successfully been developed for influenza and hepatitis A vaccines and as carrier systems for heterologous antigens for malaria, HIV or other pathogens [18]. In addition to their carrier function, virosomes possess an intrinsic immune-potentiating effect due to their surface structure consisting of incorporated influenza envelope proteins [18]. To date there is lack of knowledge on the interaction of nanocarriers like virosomes with immune cells of the respiratory tract that may provide a potential target for therapeutic immune-modulation through pulmonary administration [1]. Airway epithelial cells, alveolar macrophages and pulmonary DCs are the key players involved in maintaining barrier functions and inducing innate and adaptive immune responses. To study the interaction of nanoparticles with such cells through a more realistic approach, a human triple co-culture model with a bronchial epithelial cell line and primary MDDCs and MDMs was established and compared to respective mono-cultures [30,31,34].

Our *in vitro* triple co-culture model aims to reproduce the *in vivo* situation in humans with macrophages on the luminal side of the epithelium [35,36] and immature DCs within the lamina propria [37]. Such a model is of particular interest to investigate the ability of virosomes and liposomes to traverse the epithelial airway barrier in the respiratory tract, in which inhalable nanocarrier-based vaccines may interact with APCs such as DCs in the airway mucosa and affect downstream immune responses [38,39]. Intranasal sprays for virosome-based clinical vaccine candidates already exist [18,40] and could possibly be employed as a basis for novel therapeutic strategies with inhalable immune-modulators to treat allergic asthma.

Though MDDCs in our *in vitro* model are localized underneath the insert membrane and are therefore not directly exposed to particles upon treatment, we detected efficient and rapid uptake, especially of virosomes. It has been shown that large particles are taken up by DCs situated underneath the epithelium without affecting the epithelial cell layer integrity [41]. Several explanations are provided for this phenomenon: DCs may extend cytoplasmic processes or dendrites through epithelial tight junctions [42,43], or DCs migrate as a whole through the epithelium to capture antigen [44,45]. A study performed in a similar triple co-culture model showed that MDDCs extended long processes through the pores of the inserts to spread out directly underneath the epithelium or between epithelial cells to the apical side to capture 1 μm polystyrene particles, some MDDCs were found to have completely translocated to the apical side [2]. We assume similar cell-cell interactions in our co-cultures following exposure to virosomes or liposomes. It is of interest to mention that 16HBE cells generate a higher transepithelial electrical resistance (TEER) than the more commonly used alveolar A549 cells [14], a finding that has also been demonstrated for monolayers [46]. However, this high TEER of 16HBE does not affect particle uptake in underlying cells as we could clearly demonstrate uptake by MDDCs. Our triple co-culture model using the bronchial epithelial cell line 16HBE more closely resembles the airway than the alveolar cell line A549.

The uptake of virosomes by MDDCs is an essential result provided herein, since DCs have the ability to prime naïve T-lymphocytes [5] and regulate subsequent differentiation into effector T cells in order to modulate specific downstream immune responses [1]. Knowing that nanocarriers are able to pass the epithelial membrane and actually reach the underlying DCs is not only promising for potential therapeutic immune-modulatory strategies targeting the lung, but also for vaccination approaches to generate immunity, e.g. for respiratory syncytial virus (RSV), influenza or tuberculosis. However, optimal human *in vitro* conditions would include primary epithelial cells instead of a cell line. To investigate possible differences between epithelial cells of primary versus cell line origin, we analysed virosome and liposome uptake in parallel in both mono-cultures. Fully differentiated PHNECs were used as a surrogate cell type for bronchial epithelial cells in large airways, as it has been recently shown that readily accessible nasal epithelial cells are comparable to bronchial epithelial cells that require more invasive sampling techniques such as bronchoscopic bronchial brushings [47]. Despite the fact that PHNECs secreted more mucus than the epithelial cell line 16HBE, virosome uptake was still efficient and occurred at higher frequencies than for liposomes. Nevertheless, LSM analyses showed remaining particles in cilia and mucus on the apical side of PHNECs, explaining the lower uptake in PHNECs in comparison to 16HBE. Technically it was not possible to integrate PHNECs in the co-culture model, since primary cultures are not able to completely differentiate when grown on membranes with 3–4 μM pore-size which are required in order to provide unimpeded interactions between cells grown on either side of the membrane [48]. EpCAM^+^ cells are primary epithelial progenitor cells located in small airways [49] and were employed as a comparison to PHNECs that are also primary cells, but originate from larger airways and 16HBE, representing a cell line. With EpCAM^+^ epithelial progenitor cells we showed that both virosomes and liposomes were taken up in higher quantity per cell than by 16HBE or PHNECs. EpCAM^-^ cells, representing pericytes [27], took up significantly less virosomes compared to their EpCAM^+^ counterparts. Indeed, it was shown earlier *in vivo* that influenza virus is able to infect EpCAM^+^ epithelial progenitor cells in the lung of mice [50]. These findings highlight that pulmonary epithelial cells, as potential targets for novel therapeutic approaches employing virosomes or liposomes, display strikingly different uptake characteristics. Despite the above limitations, the established co-culture model still represents an appropriate and feasible model of the human respiratory tract airway barrier to test novel bio-mimetic nanocarrier compounds that are being developed for pulmonary administration and modulation of mucosal immune responses in the lung [30].

Overall, there was more uptake of virosomes and liposomes in APCs compared to epithelial cells, consistent with other studies employing a triple co-culture model exposed to other particles [31], despite AECs being known to be a main target for respiratory viruses such as influenza virus, rhinoviruses (RH) [51], and RSV [52]. APCs may display higher uptake than epithelial cells for various reasons: One important difference is their phagocytic activity and as macrophages are twice as phagocytic as immature DCs, they take up even more nanoparticles than MDDCs or epithelial cells [53].

The virosomal bilayer contains viral envelope proteins such as hemagglutinin and neuraminidase that both bind to sialic acid residues and trigger highly efficient receptor-mediated uptake. Liposomes, on the contrary, lack such viral proteins and thus are taken up mostly by macropinocytosis or endocytosis [54]. In co-cultures the uptake of liposomes and virosomes was generally decreased compared to mono-cultures. Previous studies showed inhibitory effects of alveolar macrophages on DC maturation both *in vivo* and *in vitro* [9,55]. Inhibition of DCs may partly be due to release of NO by macrophages and epithelial cells, which caused abrogated expression of MHC Class II [9]. However, in our model we observed a tendency of increased HLA-DR expression on MDDCs in co-culture compared to MDDCs in mono-culture. Significantly decreased particle uptake in co-cultures compared to monocultures as observed in MDDCs and epithelial cells may be due to the location of MDDCs underneath the epithelial barrier, impairing direct uptake of virosomes or liposomes administered to the upper chamber. In contrast, epithelial cells with macrophages located at their apical side may take up less particles due to clearance by the macrophages. We recently reported that polystyrene nanoparticles of different sizes administered intra-nasally in mice were mainly taken up by alveolar macrophages, as compared to DCs located in the epithelium of the airways and the alveoli, underlining the appropriateness of the present *in vitro* triple co-culture as a model system for the pulmonary epithelial barrier. In addition to HLA-DR, CD86 was increased in MDDCs following treatment with virosomes and liposomes, compared to PBS control, suggesting a moderate degree of DC activation. In contrast, MDMs treated with virosomes or liposomes showed no significant difference in phenotypic markers compared to PBS control. Though there was a tendency for upregulation of markers like CD40 in co-cultures compared to mono-cultures, this change did not reach statistical significance. Macrophage polarization associated with different respiratory disorders like asthma has received increased attention in recent years, reflecting the importance and plasticity of this population in health and disease [56–58]. We tested whether treatment with virosomes or liposomes would induce phenotype changes of macrophages that have undergone polarization. Polarization was monitored through the expression of markers HLA-DR, CD80 and CD86 for M1 and CD36, CD163 and CD206 for M2 [59]. Following treatment for 18h with virosomes or liposomes, we failed to detect any significant phenotypic changes in either macrophages population. It has previously been reported that an increased M2 differentiation occurs in allergic asthma due to a skewed Th2 immune response [57,60,61]. M1 macrophages on the other hand were associated with less severe asthma and an increase in Th1 and Th17 cells [62,63]. Therefore, it may be advantageous to shift the macrophage phenotype in asthmatic patients from a M2 type to a M1 type. It has been shown *in vitro* that differentiated macrophages can be switched back to M0 state by using specific media [64], an approach that may be of interest *in vivo*, too. Other studies suggest to treat macrophages with PGE_2_ to inhibit M2 macrophage development [56], a molecule that may be administered with virosomes and liposomes as carriers. Specifically, targeting M2 cells to transdifferentiate into a M1 phenotype may help reprogram a skewed immune balance in the lung by generating Th1-like immune response. Due to the fact that in our experiments M2 macrophages showed increased uptake of virosomes compared to M1, these nanoparticles show potential to be used as a carrier for molecules such as PGE_2_ to switch polarization of M2 macrophages. In addition to our findings, it has been shown earlier that M2 cells show higher phagocytic activity for pathogens than M1 cells [65]. The underlying mechanism might be due to high CD206 expression, a macrophage mannose receptor important for phagocytosis [66]. For this reason M2 macrophages would not need to be specifically targeted as their CD206 may be involved in internalization of different particle types.

In our co-culture experiments a moderate increase of HLA-DR and CD86 expression occurred after treatment with both virosomes and liposomes, as well as with PBS, when compared to mono-cultures. A possible explanation may be the interplay of the different cell types via cell-cell contact or the release of cytokines facilitating activation in the co-culture model compared to mono-cultures. As an example, secretion of cytokines like thymic stromal lymphopoietin (TSLP) by epithelial cells may play an important role [15]. As reviewed in Ziegler *et al*, TSLP can induce several phenotype changes on human DCs, including upregulation of MHC class II, CD40 and CD86 [16]. Furthermore, epithelial cells are able to secrete other factors that regulate DC function such as GM-CSF, IL-25, IL-33 and IL-4. In order to determine pro-inflammatory cytokine profiles, we measured IL-8 and IL-1β in supernatants from 16HBE monocultures and in the lower compartment of triple co-cultures. IL-8 is known to be secreted by epithelial cells as well as by macrophages [67] and was shown to be significantly increased in triple co-cultures compared to 16HBE mono-cultures. A similar observation was also applicable to IL-1β, which is mainly secreted by macrophages and DCs [67], both present in the triple co-culture. Müller *et al* [68] studied oxidative stress and inflammation response after nanoparticle exposure in a triple co-culture model [24] and based on their findings it is likely that the synergistic interaction of the three cell types modulate the release of cytokines and chemokines. We suggest that such a similar effect partly explains the observed increase in secretion of pro-inflammatory cytokines and upregulation of activation markers in the triple co-culture compared to mono-cultures.

In conclusion, our data underlines that the triple co-culture model provides an appropriate experimental system that realistically simulates the complexity of the airway barrier, enabling to investigate how particle-cell interactions and the interplay between different cell types affects responses to novel treatments developed for the pulmonary administration. To improve the current model, particles could be applied by means of a microsprayer or air-liquid interface cell exposure system [69]. Such an experimental setup would enable the screening of various types of derivatized virosomes and other nanoparticles for their ability to modulate immune responses in the respiratory tract, e.g. re-programming Th2-biased immune response in allergic asthma [70]. Cell-cell interactions in co-cultures affected particle uptake and release of pro-inflammatory cytokines partly reflecting the complex *in vivo* setting. The data demonstrated that *in vitro* key APCs avidly take up virosomes, yet without inducing strong activation, as measured by phenotype change or cytokine release in treated cells. This salient finding underlines the potential of such bio-mimetic particles to target immune cells in the respiratory tract, but without inducing excessive inflammatory responses that may jeopardise gaseous exchange and lung function. This characteristic makes virosomes attractive bio-mimetic nanocarriers for pulmonary antigen delivery for novel immune-modulatory strategies in the respiratory tract in disorders such as allergic asthma.

## Supporting Information

S1 Fig Cell viability after administration of nanoparticles. After incubating cells with influenza virosomes (VIRO, with (△) and without (○) Atto647), liposomes (LIPO, with (△) and without (○) Atto647) or control (PBS) for 18h, viability was tested employing a fluorescence viability dye and measuring its signal with flow cytometry. Data represents at least six independent experiments relative to PBS. Statistical significance was determined by ANOVA followed by Tukey’s HSD post hoc test to investigate individual paired comparisons.
(EPS)

S2 Fig FACS gating strategies. Cells of interest were gated according to forward and sideward scatter (FSC/SSC). Doublets were excluded by gating for single cells (FCS-W/FCS-H). Viability was determined by using two positive controls (65°C for 15 min and -80°C for 30 min). **(A)** FACS gating for triple co-culture and 16HBE cells. EpCAM^+^ cells are 16HBE cells, EpCAM^-^ cells are further divided into DC-Sign^+^ (DCs) and DC-Sign^-^ (macrophages) **(B)** Double positive CD1c^+^ and CD11c^+^ MDDCs were gated according to relevant FMO (fluorescence minus one). Phenotypic and co-stimulatory markers were analyzed according to their relevant isotype controls. **(C)** Double positive CD14^+^ and CD68^+^ MDMs were gated according to relevant FMO. Phenotypic and co-stimulatory markers were analyzed according to their relevant isotype controls.
(EPS)

S3 Fig Uptake of nanoparticles in EpCAM^-^ mono-culture. Cells were incubated with either virosomes (VIRO), liposomes (LIPO) or control (PBS) for 18h at 37°C. Uptake of virosomes and liposomes was determined by measuring Atto647 signal by flow cytometry. Frequency and MFI are shown relative to PBS. Data represents four independent experiments. Statistical significance was determined by ANOVA followed by Tukey’s HSD post hoc test to investigate individual paired comparisons.
(EPS)

S4 Fig Expression of surface markers in 16HBE cells upon uptake of nanoparticles. Cells in mono- (MO) or co-culture (CO) were incubated for 18h with either virosomes (VIRO), liposomes (LIPO) or controls (PBS, as shown). Expression of surface molecule markers HLA-DR, CD40, CD80, CD86 was measured by flow cytometry. Figures show the receptor expression in frequency of at least six independent experiments. Statistical significance was determined by ANOVA followed by Tukey’s HSD post hoc test to investigate individual paired comparisons.
(EPS)

S5 Fig Expression of surface markers and cytokines in MDDCs upon uptake of nanoparticles in mono-culture. Cells were incubated for 18h with either virosomes (VIRO, with (△) and without (○) Atto647), liposomes (LIPO, with (△) and without (○) Atto647) or controls (PBS, as shown). Expression of surface molecule markers CD83, PD-L1, PD-L2, CCR7 and intracellular cytokines IL-10 and IL-12 was measured by flow cytometry. Figures show the receptor expression in frequency **(A)** and MFI **(B)** of at least six independent experiments. Statistical significance was determined by ANOVA followed by Tukey’s HSD post hoc test to investigate individual paired comparisons. *p<0.05; **p<0.01; ***p<0.001.
(EPS)

S6 Fig Expression of cytokines in MDMs upon uptake of nanoparticles in mono-culture. Cells were incubated for 18h with either virosomes (VIRO, with (△) and without (○) Atto647), liposomes (LIPO, with (△) and without (○) Atto647) or controls (PBS, as shown). Expression of intracellular cytokines IL-10 and IL-12 was measured by flow cytometry. Figures show expression in frequency **(A)** and MFI **(B)** of at least six independent experiments. Statistical significance was determined by ANOVA followed by Tukey’s HSD post hoc test to investigate individual paired comparisons. *p<0.05; **p<0.01; ***p<0.001.
(EPS)

S7 Fig Expression of surface markers and cytokines in MDDCs upon treatment with controls in mono-culture. Cells were incubated for 18h with medium (DCs only), or positive controls LPS and inactivated virus A/Brisbane/59/2007 H1N1 (A/B). Expression of surface molecule markers HLA-DR, CD40, CD80, CD86, CD83, PD-L1, PD-L2, CCR7 and intracellular cytokines IL-10 and IL-12 was measured by flow cytometry in MDDCs. Figures show expression in frequency **(A)** and MFI **(B)** of at least six independent experiments. Statistical significance was determined by ANOVA followed by Tukey’s HSD post hoc test to investigate individual paired comparisons. *p<0.05; **p<0.01; ***p<0.001.
(EPS)

S8 Fig Expression of surface markers and cytokines in MDMs upon treatment with controls in mono-culture. Cells were incubated for 18h with medium (DCs only), or positive controls LPS and inactivated influenza virus A/Brisbane/59/2007 H1N1 (A/B). Expression of surface molecule markers HLA-DR, CD40, CD80, CD86, CD163 and intracellular cytokines IL-10 and IL-12 was measured by flow cytometry. Figures show the receptor expression in frequency **(A)** and MFI **(B)** of at least six independent experiments. Statistical significance was determined by ANOVA followed by Tukey’s HSD post hoc test to investigate individual paired comparisons. *p<0.05; **p<0.01; ***p<0.001.
(EPS)
